# Supplementary material for: T Cell Responses Correlate with Self-Reported Disease Severity and Neutralizing Antibody Responses Predict Protection against SARS-CoV-2 Breakthrough Infection
Source: Viruses. 2023 Mar 9;15(3):709. doi: 10.3390/v15030709 (PMC10058409; doi:10.3390/v15030709)
Supplement: Supplementary file 1 [file viruses-15-00709-s001.zip › viruses-2215644-supplementary.pdf]

Supplemental Materials

Supplemental Table S1. **Participants who reported previous infection and/or anti-N positive.**

		**Anti-N positive**	**Anti-N negative**	**Anti-N not measured**
**Self-reported**	previous infection	36	52	15
	no previous infection	10	555	9

Supplemental Table S2. Change in log-Biomarker over time for each vaccine type for individuals who are infection naïve and not boosted.

		Univariate			Multivariate*		
	Months (n)	Pfizer (314)	Moderna (181)	Johnson & Johnson (38)	Pfizer (314)	Moderna (181)	Johnson & Johnson (38)
NAb	0-2 (10)	0.122	-0.366	-0.581	0.053	-0.432	-1.055
	2-4 (24)	ref	ref	ref	ref	ref	ref
	4-6 (239)	**-0.505***	-0.143	**-1.069***	**-0.549***	-0.188	**-1.052***
	6-8 (193)	**-0.961*****	-0.513	-0.292	**-0.889*****	-0.537	-0.183
	8-10.5 (65)	**-1.316*****	-0.492	0.754	**-1.333*****	-0.433	0.739
Ag1	0-2 (10)	0.198	-0.061	0.076	0.187	-0.058	0.023
	2-4 (23)	ref	ref	ref	ref	ref	ref
	4-6 (236)	0.061	0.004	0.028	0.051	0.010	0.013
	6-8 (192)	0.072	0.006	0.065	0.047	0.004	0.028
	8-10.5 (66)	0.075	-0.012	0.020	0.065	-0.033	-0.004
Ag2	0-2 (10)	0.247	-0.148	0.175	0.234	-0.159	0.113
	2-4 (23)	ref	ref	ref	ref	ref	ref
	4-6 (236)	0.091	-0.045	0.101	0.073	-0.038	0.082
	6-8 (192)	0.094	-0.062	0.167	0.050	-0.069	0.107
	8-10.5 (66)	0.090	-0.037	0.040	0.069	-0.076	-0.005
Ag3	0-2 (10)	0.241	-0.165	**1.021****	0.234	-0.166	**0.987****
	2-4 (23)	ref	ref	ref	ref	ref	ref
	4-6 (236)	0.101	-0.088	0.101	0.090	-0.079	0.085
	6-8 (192)	0.108	-0.091	0.195	0.071	-0.093	0.145
	8-10.5 (66)	0.086	-0.005	-0.004	0.074	-0.038	-0.029
SAg	0-2 (10)	**0.467***	-0.296	**1.063***	**0.450***	-0.292	0.982
	2-4 (23)	ref	ref	ref	ref	ref	ref
	4-6 (236)	0.164	-0.136	0.153	0.144	-0.123	0.125
	6-8 (192)	0.163	-0.145	0.319	0.104	-0.149	0.240
	8-10.5 (66)	0.149	-0.041	0.064	0.126	-0.093	0.020

NAb=Neutralizing Antibody (U/mL), Ag1-3=T cell response to Ags 1-3 (IU/mL), SAg= sum of T cell responses to Ags 1-3 (IU/mL). Values are log assay results.

* Multivariate models include sex, age, and self-reported any preexisting conditions as covariates. Significant changes from the reference time are in bold, with '***' p<0.001 '**' p<0.01 '*' p<0.05.

Supplemental Table S3: Change in log-Biomarker at 2-4 months after complete vaccination between vaccine types for individuals who are infection naïve and not boosted. Values are log assay results. Multivariate models include sex, age, and self-reported any preexisting conditions as covariates. Significant changes from the reference time are in bold, with '***' p<0.001 '**' p<0.01 '*' p<0.05.

	**Univariate**			**Multivariate**		
**Assay**	Pfizer vs Moderna	Moderna vs J&J	Pfizer vs J&J	Pfizer vs Moderna	Moderna vs J&J	Pfizer vs J&J
**NAb**	-0.298	2.102***	1.805***	-0.341	2.232***	1.891***
**Ag1**	-0.135	0.186	0.052	-0.122	0.162	0.040
**Ag2**	-0.204	0.248	0.045	-0.181	0.212	0.031
**Ag3**	-0.255	0.236	-0.019	-0.236	0.202	-0.034
**SAg**	-0.439	0.498	0.059	-0.409	0.445	0.035
**NAb=Neutralization Antibody (U/mL), Ag1-3=T cell response to Antigens 1-3 (IU/mL), SAg= sum of T cell responses to Antigen 1-3 (IU/mL).**						

Supplemental Table S4. Risk of breakthrough infections by vaccine type or with positive (measured above LOD) test reported. Multivariate models also adjust for age, gender, time since last vaccine dose shot of blood draw, previous infection status at blood draw, and time between blood draw and infection (for comparisons of infection severity). Values are (adjusted) odds ratios for the event in the second column. Significant changes from the reference time are in bold, with '*' when p<0.05.

Univariate Analysis Results

**COVID19**	n	Moderna vs Pfizer	J&J vs Pfizer	Moderna vs J&J	Positive NAb	Positive sum Antigen	Positive Ag1	Positive Ag2	Positive Ag3
**No breakthrough**	362	1.03	0.81	1.27	1.04	1.70	1.53	1.71	1.25
**No limitations**	26	1.04	1.07	0.97	0.88	0.98	0.87	1.09	0.96
**Mild-moderate limitations**	51	1.01	1.07	0.94	1.02	1.15	1.16	0.96	1.13
**Severe limitations**	10	0.95	0.87	1.09	1.11	0.89	1.00	0.96	0.91
**Hospitalizations**	0	-	-	-	-	-	-	-	-

Multivariate Analysis Results

**COVID19**	n	Moderna vs Pfizer	J&J vs Pfizer	Moderna vs J&J	Positive NAb	Positive sum Antigen	Positive Ag1	Positive Ag2	Positive Ag3
**No breakthrough**	362	1.03	0.80	1.29	1.02	1.73	1.64	1.79	1.29
**No limitations**	26	1.06	1.12	0.94	0.97	0.97	0.87	1.02	0.94
**Mild-moderate limitations**	51	0.97	1.08	0.90	0.94	1.21	1.19	1.01	1.21
**Severe limitations**	10	0.98	0.83	1.18	1.10	0.85	0.97	0.97	0.89
**Hospitalizations**	0	-	-	-	-	-	-	-	-

Supplemental Table S5: Risk of breakthrough infections based on the status of self-reported previous infection (A) and/or positive anti-N results (B). Multivariate models also adjust for age, gender, time since last vaccine dose shot to blood draw, previous infection status at blood draw, and time between blood draw and infection (for comparisons of infection severity). Values are (adjusted) odds ratios for the event in the second column. Significant changes from the reference time are in bold, with '*' when p<0.05.

**Univariate**				
**n**	COVID19	Previous infection (A|B)	Self-reported Previous infection (A)	Positive Anti-N (B)
**362/449**	No breakthrough	0.61	0.57	0.43*
**26/87**	No limitations	1.06	1.07	0.96
**51/87**	Mild-moderate limitations	1.02	1.02	1.07
**10/87**	Severe limitations	0.92	0.92	0.97
**0/87**	Hospitalizations	-	-	-
**Multivariate**				
**n**	COVID19	Previous infection (A|B)	Self-reported Previous infection (A)	Positive Anti-N (B)
**362/449**	No breakthrough	0.61	0.57	0.58
**26/87**	No limitations	1.12	1.13	0.83
**51/87**	Mild-moderate limitations	1.01	1.00	1.11
**10/87**	Severe limitations	0.88	0.88	1.09
**0/87**	Hospitalizations	-	-	-

Supplemental Figure S1: Marginal effect of age on NAb responses and sum of T cell responses to Antigen 1-3. In the models for NAb responses, the age effect is significant ($p<0.001$), while age is not a significant predictor of the T cell responses to Antigen 1-3 ($p=0.255$).

Supplemental Figure S2: T Cell responses or levels of NAb levels in those with breakthrough infections versus months after blood draw when breakthrough infected. No significant differences of levels T cell responses or NAb were observed in those with no limitations, mild/moderate limitations, and severe limitations from symptoms due to breakthrough infections regardless the time of breakthrough infections after the blood draw. Color shows symptoms (none/mild-mod/severe limitations) during breakthrough infection. Lims: limitations; Mod: moderate

Supplemental Figure S1.

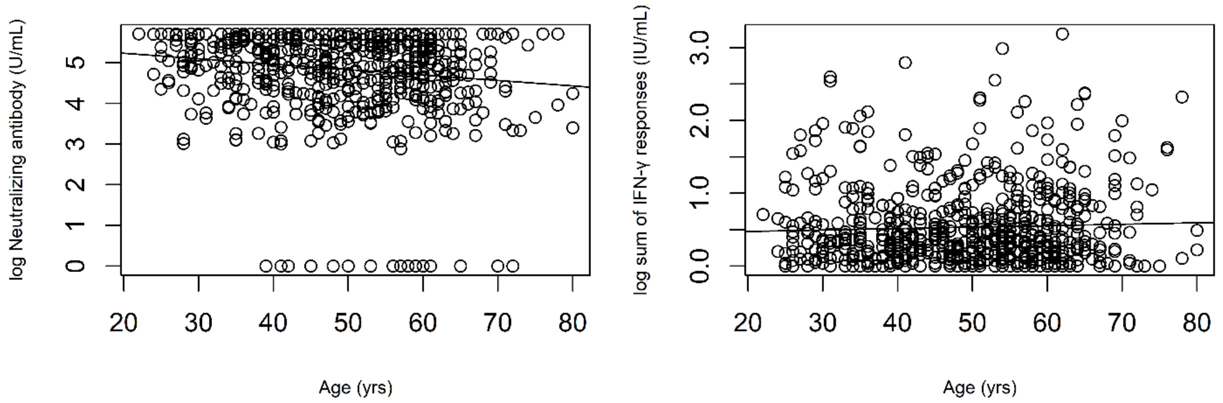

Supplemental Figure S2.

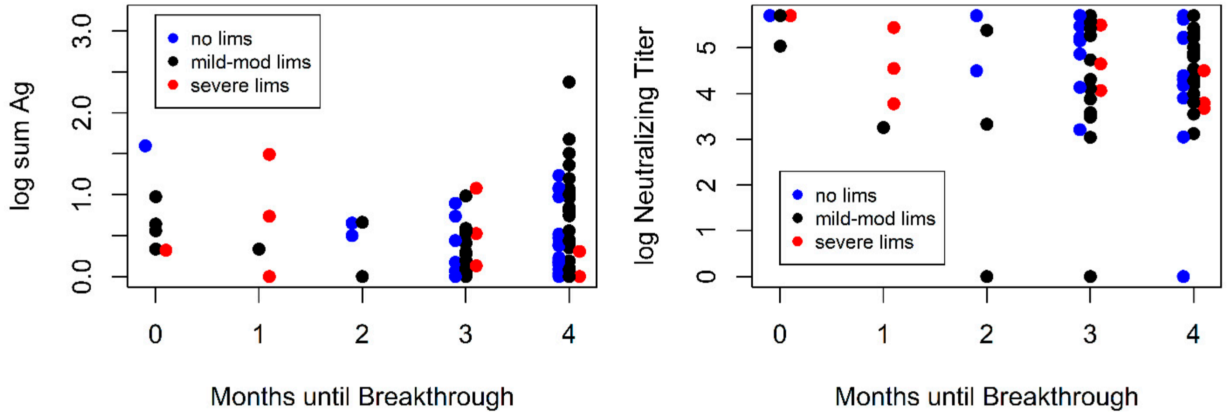

Supplemental Methods

Performance evaluation for the QuantiFERON 2 plate ELISA kit

To qualify performance of the research assay, a lithium heparin tube was collected from 47 employee volunteers at Quest Diagnostics who consented in September 2021. The employees confidentially self-reported their vaccination and/or past infection status, as having a previous diagnosis of COVID-19 and/or full vaccination defined as two doses of the Moderna or Pfizer vaccine, or one dose of the Johnson & Johnson (J&J) vaccine (n = 45), or no history of vaccination or COVID-19 infection (n = 2). Testing was performed on two Dynex Agility instruments for comparison purposes. Of the 45 volunteers that reported vaccination and/or a past diagnosed infection, 22 had antigen 1 response, 25 had antigen 2 response, and 28 had antigen 3 response. 36 of the 45 responded to at least one of the antigens. Of the two volunteers that self-reported as not having a history of vaccination or infection, both had no response for any of the three SARS-CoV-2 antigens. Kit standards were used for precision and analytical measurement range studies. The Limit of Detection (LOD) and Limit of Quantitation were established as 0.038 IU/mL and 0.061 IU/mL Interferon-gamma, respectively. LOD was applied as the cutoff value for a positive response.

QuantiFERON 2 plate ELISA kit Analysis

This assay consists of three different SARS-CoV-2 virus-specific peptide pools (antigen tubes SARS-CoV-2 Ag1, Ag2 and Ag3) to stimulate virus-specific T lymphocytes in heparinized whole blood. We also calculated SumAg, the sum of the quantitative results from antigen tubes SARS-CoV-2 Ag1, Ag2 and Ag3, to give a summary of the T lymphocyte response.

The QFN SARS CoV-2 Ag1 tube contains CD4+ T cell epitopes derived from the S1 subunit (Receptor Binging Domain) of the Spike protein, the Ag2 tube contains CD4+ and CD8+ T cell epitopes from the S1 and S2 subunits of the Spike protein, and the Ag3 tube consists of

CD4+ and CD8+ T cell epitopes from S1 and S2, plus immunodominant CD8+ T cell epitopes derived from whole genome. The control tubes include negative (Nil or no stimulant) and positive (Mitogen or non-specific stimulant) controls. Plasma from the stimulated samples was then used for detection of T cell response using the testing kit[18], as described previously [22].

Following ELISA, background INF-ɣ levels (the Nil value without stimulating peptides is background T cell reactivity in the sample that was not a result of SARS-CoV-2-specific T cell stimulation) were subtracted to obtain quantitative results (Interferon-gamma in IU/ml) for analysis.
